# Poster Session I - A55 PERFORMANCE OF LARGE LANGUAGE MODELS IN THE OPTICAL DIAGNOSIS OF COLORECTAL POLYPS

**DOI:** 10.1093/jcag/gwaf042.055

**Published:** 2026-02-13

**Authors:** J C Vences, W T Tran, N Gimpaya, C M Walsh, R Khan, R Bechara, D von Renteln, S Grover

**Affiliations:** Scarborough Health Network Centenary Hospital, Scarborough, ON, Canada; Scarborough Health Network Centenary Hospital, Scarborough, ON, Canada; Scarborough Health Network Centenary Hospital, Scarborough, ON, Canada; Paediatrics, The Hospital for Sick Children, Toronto, ON, Canada; University of Calgary, Calgary, AB, Canada; Medicine, Kingston Health Sciences Centre, Kingston, ON, Canada; Universite de Montreal, Montreal, QC, Canada; Scarborough Health Network Centenary Hospital, Scarborough, ON, Canada

## Abstract

**Background:**

Optical diagnosis allows for rapid endoscopic decision-making, but many practitioners are inadequately trained. Large language models, such as Anthropic’s Claude Opus 4, have not been evaluated in their ability to apply the NICE or JNET classification systems to colorectal polyps to more reliably predict lesion histologies. Additionally, there is a limited reference base for ideal prompting strategies in gastrointestinal endoscopy.

**Aims:**

The diagnostic accuracy of multimodal large language models (MLLMs) in classifying colorectal polyps and predicting histology will be comparable to societal (ASGE and ESGE) guidelines and to that of traditional computer-aided diagnostic systems.

**Methods:**

We conducted a retrospective diagnostic accuracy study using the PRIME dataset, a curated set of white light and narrow-band imaging (NBI) images. We evaluated Claude Opus 4, Google Gemini 2.5 Pro, GPT-o3, GPT-4o, and GPT-5. For Paris, Narrow-band Imaging Colorectal Endoscopic (NICE), and classifications and predicted histology, we calculated percent correct scores and accuracy of each MLLM compared to expert responses for 132 cases. For the Japanese NBI Expert Team (JNET) classification, we analyzed 82 cases. We calculated accuracy, sensitivity, specificity, and positive and negative predictive values. Cochran’s Q and McNemar’s Test were used to determine differences between the predicted values of each MLLM.

**Results:**

Claude Opus 4 and GPT-5 had significantly higher percent correct scores than other MLLMs at 41.7%, using Paris classification. The highest percent correct score range among all the classifications was NICE, which had a range of 78.0% to 84.1%. The accuracy scores among MLLMs were >80% for all models for neoplastic vs. non-neoplastic polyps.

**Conclusions:**

Claude Opus 4 and Gemini 2.5 Pro showed the highest accuracy in differentiating colorectal polyp subtypes, performing closest to expert consensus. Sensitivity and specificity, however, did not meet ESGE standards, highlighting the need for prospective multicenter trials and the design of human-in-the-loop workflows before clinical deployment.

**Funding Agencies:**

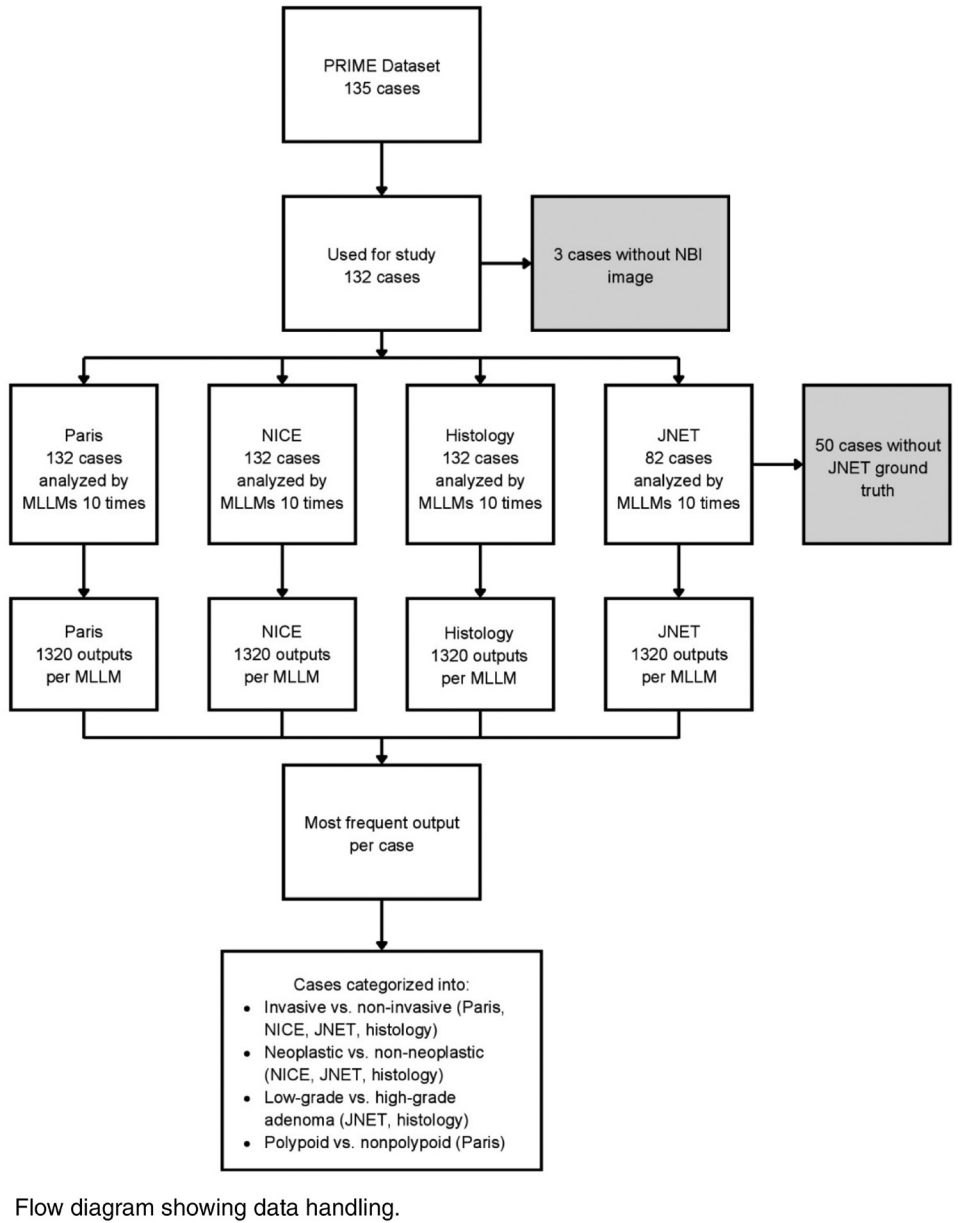

Pialis Family Chair in Education

**Table:** 

	Paris (n = 132)		NICE (n = 132)		JNET (n = 82)		Histology (n = 132)	
MLLM	Correct (%)	Cochran’s Q (p-value)	Correct (%)	Cochran’s Q (p-value)	Correct (%)	Cochran’s Q (p-value)	Correct (%)	Cochran’s Q (p-value)
Claude Opus 4	41.7^a^	32.9 (<0.001)	84.1	4.48 (0.34)	59.8	1.19 (0.88)	62.1	4.19 (0.38)
Gemini 2.5 Pro	30.3^b^		78.8		58.5		55.3	
GPT-o3	33.3^c^		78.0		56.1		60.6	
GPT-4o	24.2^d^		80.3		54.9		60.6	
GPT-5	41.7^e^		78.8		54.9		62.9	

Pairwise comparisons showed statistical difference (p < 0.05) based on McNemar’s test (a) with Gemini 2.5 Pro, GPT-o3, and GPT-4o; (b) with Claude Opus 4 and Gemini 2.5 Pro; (c) with Claude Opus 4, GPT-4o, and GPT-5, (d) with Claude Opus 4, GPT-o3, and GPT-5; (e) with Gemini 2.5 Pro, GPT-o3, and GPT-4o.

